# A Scheme for System Error Calibration and Compensation of the Initial State of MEMS Inertial Navigation

**DOI:** 10.3390/s25216668

**Published:** 2025-11-01

**Authors:** Xiangru Ding, Zhaobing Chen, Zhaolong Wu, Xiushuo Wang

**Affiliations:** 1 University of Chinese Academy of Sciences, 1 Yanqihu East Rd, Huairou District, Beijing 100049, China; dingxiangru23@mails.ucas.ac.cn (X.D.); wangxiushuo22@mails.ucas.ac.cn (X.W.); 2 Changchun Institute of Optics, Fine Mechanics, and Physics, Chinese Academy of Sciences, 3888 DongNanhu Road, Changchun 130033, China; wuzhaolong20@mails.ucas.ac.cn

**Keywords:** MEMS, inertial navigation, system-level calibration, initial alignment

## Abstract

Aiming at the challenge of balancing the accuracy and cost of the initial state calibration of traditional MEMS inertial navigation systems, as well as the current situation of the lack of high-precision three-axis turntables in engineering practice, this paper proposes a practical and innovative systematic error calibration and compensation scheme, which effectively suppresses the deterministic errors of MEMS-INS and enhances its applicability in high-precision and long-duration tasks. By analyzing the coordinate transformation characteristics of the MEMS-INS solution process under small-angle disturbances, a deterministic error model based on the device’s zero bias, scale factor, and cross-coupling errors is constructed. A twelve-position dual-axis calibration method, combined with a high-precision orthogonal fixture, is designed to excite errors on a dual-axis turntable, converting originally unobservable error terms into observable periodic signals. Experimental results show that the installation error calibration accuracy reaches 0.03°, an improvement of about 25% compared to the traditional dual-axis method, breaking through the limitations of dual-axis turntables in cross-coupling error calibration, achieving an initial error ≤ 1 μrad, and reducing the navigation error by 90% within one hour. This method eliminates reliance on expensive three-axis turntables while enabling multi-error calibration, addressing the cost–accuracy trade-off in engineering applications.

## 1. Introduction

The inertial navigation system (INS) is based on Newtonian mechanics [1]. By high-frequency sampling of the angular increments and specific force information output by gyroscopes and accelerometers, via strapdown configuration and integration, it can recursively calculate the real-time attitude, velocity, and position of the carrier. These deterministic errors accumulate quadratically or cubically during the integration process, seriously limiting the applicability of MEMS-INS in high-precision, long-duration missions [2].

The core value of initial state calibration lies in providing high-precision initial conditions for navigation solutions [3]. It not only requires accurate determination of the carrier’s initial position, velocity, and attitude but also demands estimation of and compensation for the systematic errors of inertial devices (e.g., zero bias and scale factor errors of gyroscopes and accelerometers). Any deviation in the initial state will continuously amplify as the navigation time elapses, directly impairing the overall accuracy of the system. In contrast, precise initial calibration can effectively enhance the stability and reliability of MEMS-INS in complex environments and mitigate the risk of navigation failure caused by sensor errors.

It is one of the most cost-effective means to improving the accuracy of a micro-electromechanical system–inertial navigation system (MEMS-INS) by introducing periodic attitude changes through rotation modulation technology and transforming the inertial device errors into periodic signals with observable or mean zero. According to the number of rotation axes, the technology is mainly divided into three categories, single-axis, double-axis and three-axis rotation, and their application characteristics and effects are significantly different.

The core of single-axis rotation is to make the Inertial Measurement Unit (IMU) rotate around a single axis periodically, focusing on the calibration of the horizontal error perpendicular to the rotation axis. In specific scenarios, the optimization strategy can be used to improve the calibration effect. In terms of error optimization, ref. [4] quantifies the coupling relationship between the angular rate and deterministic error by Laplace transform and proposes an angular rate optimization scheme: when the gyro zero bias is large, the high angular rate is selected to enhance the zero bias suppression effect. When the scaling factor error is significant, a low angular rate is used to avoid error amplification. Aiming at the calibration problem in dynamic scenarios, ref. [5] points out that the traditional single-axis “static four-position start-stop” method can easily fail when the carrier has installation errors. To solve this problem, a dwell time adjustment strategy based on heading angle integration is proposed. Finally, the installation error calibration accuracy in dynamic scenes is improved by 40%. However, uni-axial rotation has inherent limitations: it can only stimulate the horizontal error perpendicular to the rotation axis and cannot calibrate the deterministic error along the rotation axis. At the same time, the eccentricity of the shaft system of the single-axis turntable will introduce additional deterministic errors, which need to be calibrated separately to reduce the IMU installation error deviation.

By rotating the IMU around two orthogonal axes alternately, dual-axis rotation can realize the calibration of all horizontal errors and some vertical errors. It is the mainstream scheme for medium- and high-precision inertial navigation scenarios and has led to a number of key breakthroughs in error modulation and optimization. Ref. [6] aimed at the defect of “installation error leads to speed accumulation” in the traditional 8/16 position scheme and designed a 16-position sequence of “external axis bidirectional rotation + internal axis rotation”, which modulated the IMU rotation axis error from non-zero mean to zero mean, effectively reducing the error peak-to-peak value. At the same time, the non-orthogonal angle of the shaft system is calibrated by moving forward and reverse alternately to further improve the accuracy. In reference [7], the Schuler period was used to suppress the unconstrained propagation of position, and the Schuler period optimization method for the scaling factor error was proposed: by adjusting the rotation sequence, the mean value of the scaling factor error of the horizontal gyro was zero in the period, and the scaling factor error was reduced from 20 ppm to 5 ppm after calibration. It should be noted that the bi-axial rotation can only effectively excite the error term perpendicular to the rotation axis, and it is not sensitive enough to the error along the rotation axis. Moreover, during the rotation process, the IMU installation error will be coupled with the rotation angular velocity, resulting in a periodic attitude error. 

The three-axis rotation inertial navigation system can more comprehensively excite various errors of inertial devices, including gyro-scale factor errors, installation errors, alignment errors, and accelerometer non-orthogonal errors, thereby achieving more accurate calibration of inertial devices’ deterministic errors [8]. Through precise measurement and analysis of the inertial device output during the three-axis rotation process, a more complete error model can be established, providing a more reliable error compensation basis for high-precision navigation solutions. Ref. [9] proposes a 36-dimensional Kalman filter system-level calibration method, which simultaneously estimates the zero bias, scale factor, installation error, quadratic coefficient, and internal boom arm parameters of the gyros and accelerometers. By designing a calibration route including multi-axis rotation and oscillation, researchers have significantly improved the calibration accuracy in complex environments. Ref. [10] proposes an outer longitudinal–middle roll–inner heading three-axis nested structure, using PID control of the outer axis to isolate the carrier heading disturbance and combining the sixteen-position dual-axis modulation to achieve full-degree-of-freedom error suppression. The mechanical structure of the three-axis system is precise, and currently, the manufacturing cost of such equipment is high, and the manufacturing difficulty is significant. At the same time, the three-axis inertial navigation system generates a large amount of data and has a complex error model. To achieve precise estimation and compensation of inertial devices, high hardware performance and algorithm requirements are necessary for the system.

Considering the constraints of engineering applications, this paper focuses primarily on the installation of inertial navigation systems behind airborne electro-optical turntables and designs an initial error calibration method using the IMU as the core device for positioning scenarios. Given that high-precision three-axis turntables are scarcely available in engineering practice, a twelve-position dual-axis calibration scheme is proposed to maximize the calibration of various existing error coefficients, with the rotation principle being simplified for practical engineering applications.

The article is structured as follows: The first section briefly introduces the accuracy limitations of MEMS inertial navigation in current applications and analyzes relevant initialization and calibration methods. The second section identifies and analyzes potential error factors throughout the entire data acquisition and computation process, based on the practical application of inertial navigation. The third section designs an initial calibration scheme tailored to these error factors. The fourth section verifies the effectiveness of the proposed method through experiments and simplifies it to enhance its practicality in engineering applications. Finally, the research findings are summarized, and future improvement directions are proposed.

## 2. Analysis of the Working Principle and Error Sources of Inertial Navigation

### 2.1. Error Propagation Analysis of Inertial Navigation

#### 2.1.1. Definition of the Coordinate System

In an aircraft-mounted inertial navigation system (INS), inertial sensor data (from gyroscopes and accelerometers) must be transformed through a series of coordinate systems to map measurements from the local device frame to the global Earth reference frame. This transformation process enables the INS to compute attitude, velocity, and position. The sensors on the carrier generate raw data in the MEMS-based device coordinate system (g-system). Due to mechanical misalignments, installation errors occur when transforming from the g-system to the body coordinate system (b-system). These, along with inherent sensor errors such as gyroscope bias and accelerometer scale factor inaccuracies, are corrected through calibration. Next, the b-system is converted to the navigation coordinate system (n-system) using the attitude direction cosine matrix (DCM), aligning with the carrier’s motion reference [11]. Finally, the n-system is mapped to the Earth coordinate system (e-system), typically the Earth-centered Earth-fixed (ECEF) frame, via the position correlation matrix. This last step supports integration with external systems like GNSS or BeiDou by standardizing the reference frame and reducing positional mismatches. For accurate error analysis, the transformation sequence is as follows: g-system → b-system → n-system → e-system.

**Figure 1:**
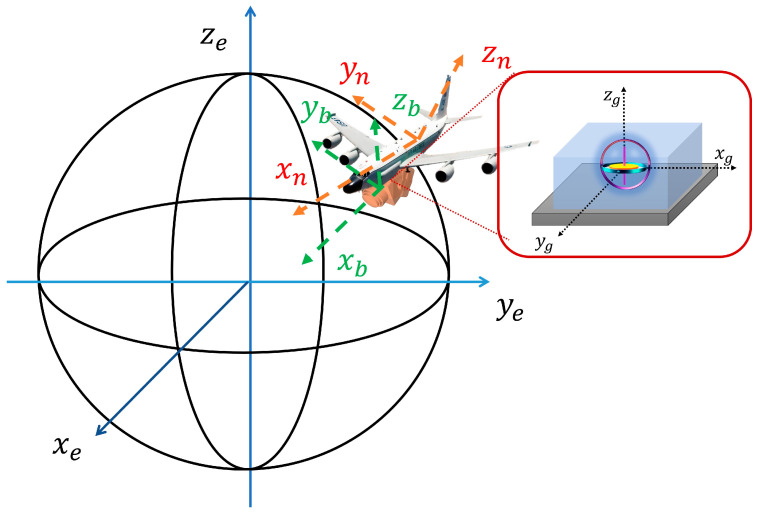
Coordinate transformation diagram.

1. Establish the MEMS inertial navigation three-axis measurement coordinate system (system g), with the origin being located at the rotation center. The three axes are set according to the actual data manual of the gyroscope. The schematic diagram is shown in Figure 1.
2. Establish that the carrier coordinate system (system b) is based on the load platform as the reference. Let the origin of this coordinate system be located at a certain fixed point on the load platform. The $x_{b}$ axis runs along the longitudinal direction of the load platform (pointing in the direction of movement), the $y_{b}$ axis runs along the transverse direction of the load platform, and the $z_{b}$ axis is perpendicular to the load platform and points upwards.
3. The navigation coordinate system (n system) adopts the Northeast-Up (ENU) coordinate system. The $x_{n}$ axis points eastward, the $y_{n}$ axis points northward, and the $z_{n}$ axis points towards the zenith.
4. Geocentric geodetic coordinate system (e system): The origin is located at the center of the Earth. The $x_{e}$ axis passes through the equatorial point of the prime meridian, the $y_{e}$ axis passes through the equatorial point of 90° east longitude, and the $z_{e}$ axis runs along the Earth’s rotational axis (the North Pole).

#### 2.1.2. Coordinate Transformation Analysis

The transformation between multiple coordinate systems is achieved through the direction cosine matrix (DCM). Let $C_{j}^{i}$ be the DCM from system j to system i, while its element $C_{j}^{i}(l,k)$ represents the cosine of the angle between the k-axis of system j and the l-axis of system i. 

According to the coordinate system transformation sequence, the transformations between the four coordinate systems are analyzed, which satisfy the following relational expression [12]:

(1) $$C_{e}^{g}=C_{e}^{n}\cdot C_{n}^{b}\cdot C_{b}^{g}$$

In the formula, matrix $C_{b}^{g}$ represents the transformation matrix from system g to system b, reflecting the installation relationship between the device and the carrier.

Matrix $C_{n}^{b}$ represents the transformation from system b to system n, reflecting the attitude of the carrier, and is determined by the roll angle γ, pitch angle θ, and heading angle φ.

Matrix $C_{e}^{n}$ represents the transformation matrix from system n to system e, reflecting the position of the navigation system in the Earth coordinate system, which is determined by latitude L and longitude λ.

The mathematical expression of the transformation matrix in the ideal state is as follows:

(2) $$C_{b}^{g}=I=\left[\begin{array}{ccc} 1 & 0 & 0\\ 0 & 1 & 0\\ 0 & 0 & 1 \end{array}\right]$$

(3) $$C_{n}^{b}=\left[\begin{array}{ccc} \cos\theta \cos\varphi & \cos\theta \sin\varphi & -\sin\theta \\ \sin\gamma \sin\theta \cos\varphi -\cos\gamma \sin\varphi & \sin\gamma \sin\theta \sin\varphi +\cos\gamma \cos\varphi & \sin\gamma \cos\theta \\ \cos\gamma \sin\theta \cos\varphi +\sin\gamma \sin\varphi & \cos\gamma \sin\theta \sin\varphi -\sin\gamma \cos\varphi & \cos\gamma \cos\theta \end{array}\right]$$

(4) $$C_{e}^{n}=\left[\begin{array}{ccc} -\sin\lambda & \cos\lambda & 0\\ -\sin L\cos\lambda & -\sin L\sin\lambda & \cos L\\ \cos L\cos\lambda & \cos L\sin\lambda & \sin L \end{array}\right]$$

### 2.2. Classification and Modeling of Inherent Errors of Devices 

MEMS inertial devices include gyroscopes and accelerometers [13]. The physical characteristic defects of these devices themselves are the core sources of INS deterministic errors. Based on the influence mechanism of errors on measurement values, they can be classified into three categories: zero bias, scale factor error, and cross-coupling error. The main influencing factors are shown in Table 1 below.

The zero bias of a gyroscope generally stems from temperature drift, circuit noise, and mechanical stress release, which can affect the accuracy of angular velocity transfer from the G-frame to the B-frame. Considering the zero drift and the random noise $\varepsilon_{g}$ that the gyroscope is subjected to, the measurement value of the gyroscope based on the G-frame can be expressed as follows:

(5) $$\omega_{mea}^{g}=\omega_{true}^{g}+b_{g}+\varepsilon_{g}$$

Due to the uneven manufacturing process, there is a force bias between the gyroscope output and the true angular velocity. The actual scale factor is expressed as follows:

(6) $$K_{g,act}=K_{g,nom}(1+\delta K_{g})$$

The cross-axis coupling error stems from the non-orthogonality of the shaft system. The gyroscope of one axis will be disturbed by the angular velocities of other axes. The expression of the cross-axis coupling error is analyzed and established as follows:

(7) $$S_{g}=\left[\begin{array}{ccc} 0 & S_{gxy} & S_{gxz}\\ S_{gyx} & 0 & S_{gyz}\\ S_{gzx} & S_{gzy} & 0 \end{array}\right]$$

The corrected measurement value is established as follows:

(8) $$\omega_{mea}^{g}=K_{g}(\omega_{true}^{g}+S_{g}\cdot \omega_{true}^{g})+b_{g}+\varepsilon_{g}$$

The accelerometer also has the problem of sensitive components. Compared with the gyroscope, the cross-axis coupling error does not need to be considered. Combining the analysis process of the gyroscope, the measured value of the accelerometer is corrected. The mathematical expression is as follows:

(9) $$a_{mea}^{g}=K_{a,act}\cdot a_{true}^{g}+b_{a}+\varepsilon_{a}$$

### 2.3. Error Propagation and Modeling in the Inertial Navigation Solution Process

The initial alignment error of MEMS INS is usually less than 1°, and the second-order and higher-order small terms can be ignored. The coordinate transformation error is described by a small-angle skew-symmetric matrix.

Let the small-angle error vector be ϕ.

(10) $$\phi ={\left[\begin{array}{ccc} \phi_{x} & \phi_{y} & \phi_{z} \end{array}\right]}^{T}$$

The corresponding skew-symmetric matrix ${\left[\phi \right]}^{\times }$ is defined as follows:

(11) $${\left[\phi \right]}^{\times }=\left[\begin{array}{ccc} 0 & -\phi_{z} & \phi_{y}\\ \phi_{z} & 0 & -\phi_{x}\\ -\phi_{y} & \phi_{x} & 0 \end{array}\right]$$

In practical applications, the ideal relationship of coordinate system transformation is disrupted by errors. During the transformation from system g to system b, the installation deviation of MEMS devices and the carrier causes a small angular error of system g relative to system b. Currently, the actual transformation matrix is no longer a unit matrix but is expressed as follows:

(12) $$C_{b}^{g}=I+{\left[\phi_{gb}\right]}^{\times }$$

(13) $$\phi_{gb}={\left[\begin{array}{ccc} \phi_{gbx} & \phi_{gby} & \phi_{gbz} \end{array}\right]}^{T}$$

Similarly, in the other two coordinate system transformation processes, small angle errors will be produced due to measurement deviations.

The initial state error of INS directly determines the subsequent navigation accuracy. Based on the error sources analyzed above, the error equations of the initial position, velocity, and attitude are established.

Initial position error equation:

(14) $$\delta P_{e}(0)=C_{e}^{n}\cdot \delta P_{n}(0)-{\left[C_{e}^{n}\cdot P_{n}(0)\right]}^{\times }\cdot \phi_{ne}(0)$$

Here, $P_{e}$ represents the position in the e system, and $P_{n}$ represents the position in the n system.

Initial velocity error equation:

(15) $$\delta {\dot{v}}_{n}(0)=C_{b}^{n}\cdot \delta a_{b}(0)+{\left[C_{b}^{n}\cdot \delta a_{\;\;bi}^{b}(0)\right]}^{\times }\cdot \phi_{bn}(0)-2{\left[\omega_{ie}^{n}(0)\right]}^{\times }\cdot \delta v_{n}(0)$$

Here, $\delta a_{b}$ is the projection of the accelerometer measurement error in the b coordinate system. $a_{bi}^{b}$ represents the acceleration of the body in the b system relative to the inertial system. $\omega_{ie}^{n}$ represents the projection of the Earth’s rotational angular velocity in the n coordinate system.

Initial attitude error:

(16) $${\dot{\phi }}_{bn}(0)={\left[\omega_{bn}^{b}(0)\right]}^{\times }\cdot \phi_{bn}(0)-(\delta \omega_{g}^{b}(0)+{\left[\phi_{gb}\right]}^{\times }\cdot \omega_{bn}^{b}(0))$$

In summary, the initial error of INS originates from three aspects: First, there is an installation error between the g system and the b system. Second, there are inherent errors in the device itself. The third is the distortion of coordinate system transformation. The cross-coupling error and installation error are the bottlenecks that mean that the traditional dual-axis turntable cannot be effectively calibrated. The calibration scheme designed in Section 3.2 will focus on solving the above problems.

## 3. Design of the Initialization Error Calibration Experimental Scheme

### 3.1. Initialization and Calibration Principles

Based on the above analysis, in the initial stage of inertial navigation, the deterministic errors of the equipment and the coordinate transformation during the application process can be calibrated. This paper mainly focuses on the calibration schemes of inertial navigation in practical engineering applications, assuming that the inertial navigation equipment has undergone high-precision calibration during the design and manufacturing stages. Therefore, this scheme mainly addresses the work in the engineering assembly and debugging stages and the degradation of MEMS devices after a period of operation [14]. Due to cost constraints and the limitations of the experimental environment, high-precision calibration turntables are often unobtainable. Current research shows significant progress in the design of high-precision calibration algorithms; thus, this paper proposes a simple yet effective rotational modulation scheme. Through the twelve-position rotational calibration method, the system can maximize calibration using existing equipment, thereby overcoming the inability of single-axis rotation to measure cross-axis coupling errors. The core principle of dual-axis rotational calibration is to convert previously unobservable error terms (e.g., installation errors and cross-coupling errors) into observable periodic signals by coordinating the rotation of the inner frame (pitch axis) and outer frame (azimuth axis), followed by estimating error parameters via filtering algorithms. Dual-axis rotational initial calibration suppresses deterministic errors of the inertial navigation system at the source through dynamic excitation and parameter identification, making it a key technology for balancing the low cost of MEMS devices and high navigation accuracy. Despite the lack of high-precision equipment in engineering applications, effective error compensation can still be achieved in low-cost scenarios by optimizing trajectory design, introducing system constraints, and simplifying parameter sets.

### 3.2. Design of Initialization Calibration Method

**Figure 2:**
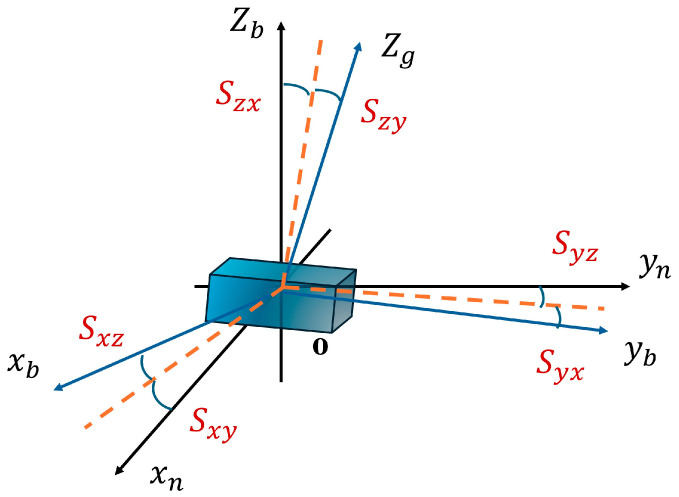
Diagram showing a shaft coupling error.

The theory of cross-axis coupling error is represented by coordinate axes, as shown in Figure 2. The deterministic error model constructed in this paper estimates the installation error as one of the core parameters. This means that the model can not only calibrate the inherent errors of inertial devices but also compensate for the installation errors introduced by minor manufacturing deviations of the fixture or long-term wear. Through regular system-level calibration, these parameters can be re-estimated and updated, ensuring that the method maintains long-term stable calibration performance even when the fixture undergoes slow degradation.

The deterministic error model of the system is integrated as follows:

(17) $$\omega_{mea}=(I+\delta K)\omega_{true}+S\omega_{true}+\varepsilon_{0}$$

In the formula, $\omega_{mea}={\left[\begin{array}{ccc} \omega_{mea-x} & \omega_{mea-y} & \omega_{mea-z} \end{array}\right]}^{T}$ represents the measured output angular velocity of the inertial navigation system, while $\omega_{ture}={\left[\begin{array}{ccc} \omega_{ttue-x} & \omega_{true-y} & \omega_{true-z} \end{array}\right]}^{T}$ represents the input angular velocities on the three axes of the inertial navigation system, which are obtained using more precise measuring instruments.

**Figure 3:**
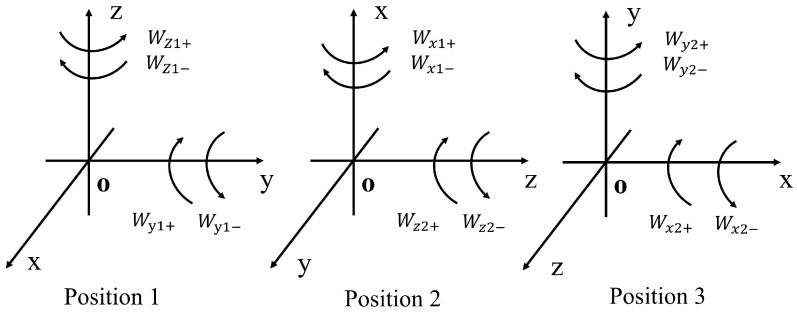
Diagram showing the rotation direction of the twelve-position mechanism.

In practical engineering applications, there is usually no high-precision three-axis measurement turntable used in the laboratory. Therefore, a three-position calibration scheme using a dual-axis turntable rotation was designed, which has higher universality. At the same time, this scheme can eliminate the influence of gravity and the Earth’s rotation on the gyroscope. The twelve-position rotation is shown in Figure 3 below.

At the beginning of each position, the output speed when the rotation axis is in a stationary state for 30 s needs to be recorded. Then, 10 sets of input angular velocities are set: ±5°/s, ±10°/s, ±15°/s, ±20°/s, and ±25°/s. The sampling time for each individual test is set to 30 s, and the default sampling rate is (400/s). Finally, a 30-s record of the stationary state after rotation is made. After testing each position, the system needs to be turned off for cooling, then reconnected to the computing rod, and the data should be saved separately and named.

Through the speed experiment, researchers can calculate the three parameters for each of the inertial navigation system’s three axes, namely the scale factor, the fitted zero position, and the nonlinearity of the scale factor. For each input speed, the average output angular velocities for forward and reverse directions are calculated, respectively, and are denoted as ${\bar{W}}_{ij}+(i=1.2\ldots ,j=x.y.z)$ and ${\bar{W}}_{ij}-(i=1.2\ldots ,j=x.y.x)$.

At the same time, we can calculate the average output in the stationary state before and after rotation and denote them as ${\bar{W}}_{s}$ and ${\bar{W}}_{e}$, respectively.

The final average output angular velocity is expressed as follows:

(18) $$W_{i}={\bar{W}}_{i}-\frac{1}{2}({\bar{W}}_{s}+{\bar{W}}_{e})$$

Calculate the output angular velocity for each position and each axis in a single rotation direction separately (as the 12 rotational angular velocities shown in the figure):

(19) $$W_{j}=\frac{\sum_{i=1}^{n}W_{i}}{n}$$

By using the turntable to rotate the sensitive axis of the gyroscope at different known angular velocities $\omega_{i}$, theoretically, the gyroscope outputs an electrical signal $V_{i}$ that is proportional to the angular velocity. Through conversion, the output angular velocity $W_{i}$ along the axial direction of the gyroscope can be obtained. In an ideal situation, the output signal $W_{i}$ satisfies a linear relationship with the input angular velocity $W_{i}=K\omega_{i}$. By setting a series of different rotation angular velocities, $\omega_{1},\omega_{2}\ldots \omega_{n}$, on the turntable and measuring the corresponding output signals, $W_{1},W_{2}\ldots W_{n}$, of the gyroscope, these data are then linearly fitted to obtain the actual proportion factor, $K_{ture}$, and compared with the nominal proportion factor $K_{mea}$ of the gyroscope to determine the proportion factor error $\Delta K=K_{ture}-K_{mea}$.

Linear fitting of the proportion factor K:

(20) $${\bar{W}}_{mea}=K_{j}W_{ture}+\varepsilon_{j}$$

Ideally, after eliminating the zero bias, the theoretical output should be $K_{j}W_{ture}$.

The zero bias error $\varepsilon_{0}$ is calculated as follows:

(21) $$\varepsilon_{0}=\frac{1}{K}\sqrt{\frac{1}{n-1}\sum_{i=1}^{n}{\left(W_{i}-\bar{W}\right)}^{2}}$$

Least squares method for solving installation errors S:

(22) $$S={\left(H^{T}H\right)}^{-1}H^{T}W_{mea-cross}$$

Here, H is the coefficient matrix constructed based on the input angular velocity, and $W_{mea-cross}$ represents the cross-talk output of the non-diagonal axes.

## 4. Experimental Verification and Data Processing

**Figure 4:**
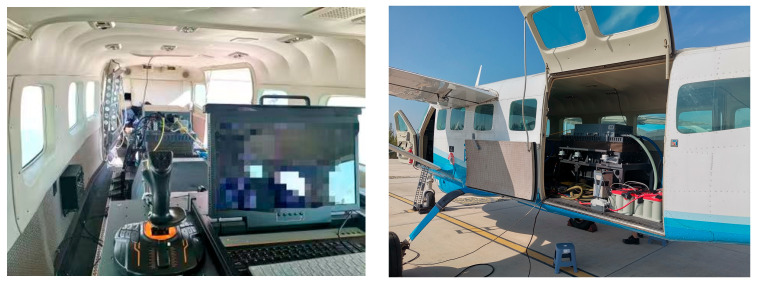
Photo of the experimental testing site.

**Figure 5:**
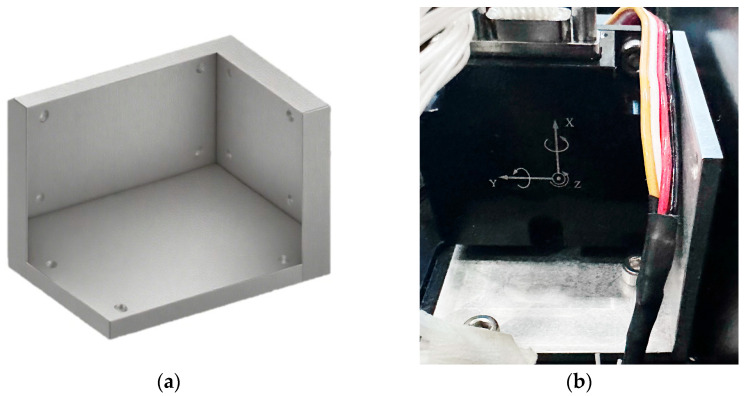
(**a**) Schematic diagram of high-precision orthogonal fixture model; (**b**) workpiece measurement installation drawing.

This experiment employs the Lord Microstrain 3DM-CV7-INS model of MEMS inertial navigation. The hardware of this navigation system mainly consists of three MEMS gyroscopes with zero bias stability of 1.5°/h and three quartz accelerometers with zero bias stability of less than 1 mg. In the research, it mainly cooperates with the airborne electro-optical turntable to complete the positioning work. The high-precision photoelectric encoder is used to synchronously measure the rotational angular velocity as the experimental true value. In this experiment, the PTN-1-type absolute encoder is selected. This product is manufactured by China Yuheng Optical Company. It operates at a supply voltage of 5 V and requires a working current of less than 350 mA. It can work in an environment with a temperature range of −40 to 70 °C and a relative humidity of ≤90% RH. It can adapt to vibrations of 55 to 2000 Hz and ≤300 m/s^2^, as well as impacts of ≤1000 m/s^2^ and 6 ms. Due to engineering limitations, the azimuth angle range of the electro-optical turntable used is 360°, and the pitch angle range is −10° to 70°. Combining practical and theoretical designs, in the actual experiment, when rotating and measuring at each position (Position 1, Position 2, Position 3), the azimuth axis is set to rotate at 10°/s, 20°/s, 30°/s, 40°/s, and 50°/s in sequence. The measurement time is set to remain stationary for 30 s, then rotate forward for 30 s, followed by remaining stationary for another 30 s, and then rotate backward for 30 s. During the movement of the azimuth axis at different speeds, the pitch axis is set to perform sinusoidal oscillations at different frequencies and amplitudes, so that the theoretical mean value of the pitch axis measurement tends to zero. The physical diagram of the experimental device is shown in Figure 4. In the experiment, to achieve the three-position setting, a high-precision orthogonal three-position workpiece was designed, as shown in Figure 5. The orthogonality of the fixture was calibrated using a three-coordinate measuring machine and a laser interferometer to ensure that its dimensional fluctuation was less than 0.002 mm and to avoid introducing new installation errors [15]. Considering the universality of engineering applications, in practical promotion, a strategy combining “precision machining and post-calibration” can be adopted. Firstly, high-precision rough machining is carried out using general equipment such as CNC, and then the actual orthogonality of the fixture is precisely measured with a three-coordinate measuring machine (CMM) or a laser interferometer. The measured deviation values are then input as known parameters into the subsequent error model for compensation. This approach can effectively reduce the extreme reliance on a single mechanical processing step and enhance the reproducibility of the method in general industrial environments.

**Figure 6:**
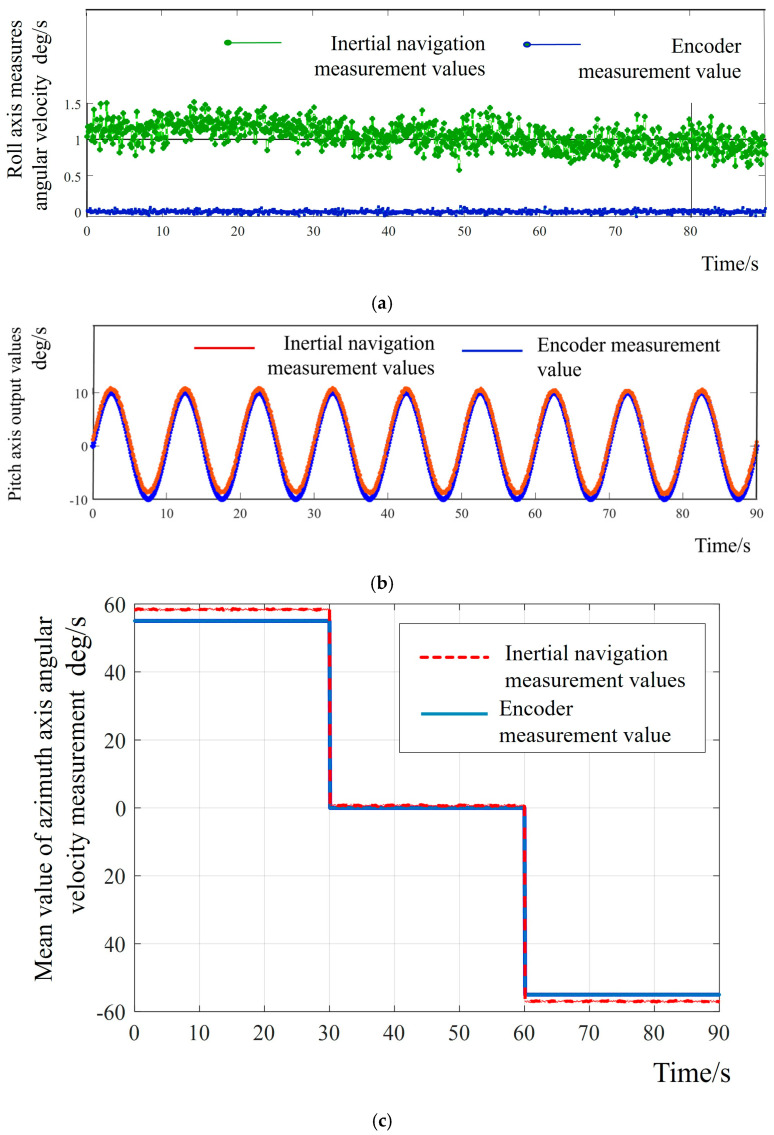
(**a**) Comparison of roll axis angular velocity accuracies; (**b**) comparison of pitch axis angular velocity accuracies; (**c**) comparison of azimuth axis angular velocity accuracies.

The three-axis data of the turntable obtained through rotation measurement in the manner of Position 1 are shown in Figure 6. Based on this, the errors existing between the X-Y-axes in the inertial navigation can be analyzed. The zero offset and proportional factor errors of the shaft system obtained through data processing and analysis are shown in Table 2.

Based on the relevant tests at location 1, the data measurements at location 2 and location 3 were repeated. After three different position measurements and data processing, the cross-axis coupling error S of the turntable rotation and inertial navigation measurement could be obtained.

(23) $$S=\left[\begin{array}{ccc} 0 & 0.0023 & 0.002\\ 0.9934 & 0 & 0.1683\\ 1.0066 & 0 & 0 \end{array}\right]$$

**Figure 7:**
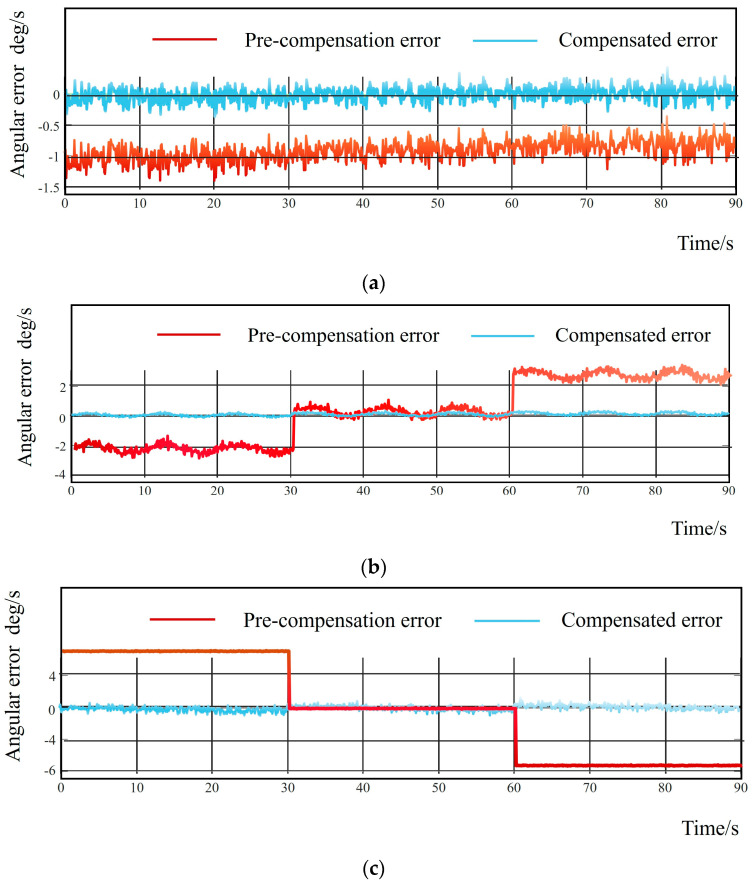
(**a**) Roll angle filtering error before and after filtering; (**b**) error before and after azimuth filtering; (**c**) error before and after pitch angle filtering.

Based on the aforementioned information, an error model for the initial state of the inertial navigation system can be established to compensate for the initial positioning errors. By integrating this model with the precise alignment filtering algorithm [16], the actual output data of the airborne electro-optical turntable in practical applications can be effectively measured. A design verification experiment was conducted to evaluate the output angular velocity of the airborne electro-optical turntable after a sequence of movements: repeating the aforementioned rotation–stationary sequence (Figure 7). The results clearly demonstrate a significant improvement in the accuracy of the compensated output data.

As shown in Table 3, the standard deviation of the measured values after compensation relative to the true values can also be observed to achieve a good result.

To evaluate the robustness and repeatability of the proposed calibration method, statistical analysis was conducted on the key performance indicators. All experiments were repeated n = 50 times under the same conditions, and the mean, standard deviation, and 95% confidence interval were calculated. The mean calibration accuracy of installation errors for the traditional two-axis method is 0.040°. The proposed twelve-position two-axis calibration method in this paper has a mean calibration accuracy of installation errors of 0.030° ± 0.005° in 50 repeated experiments, which is a 25% improvement. The within-group standard deviation of this result is 0.0021°, and the calculated 95% confidence interval is [0.0291°, 0.0309°]. A paired-sample t-test shows that the performance improvement is statistically significant compared to the traditional method (*p* < 0.001).

The average error reduction rate of the system navigation before and after compensation was 90.2%, based on 10 one-hour navigation solution tests, each repeated 50 times, with a standard deviation of 1.5% and a 95% confidence interval of [89.1%, 91.3%]. This result demonstrates that the method has high consistency and effectiveness in suppressing error accumulation. The above-mentioned repetitive experiments were completed by different operators on different working days over a period of more than one week. The fact that all key performance indicators showed no significant difference (*p* > 0.05) among different experimental batches proves that this calibration scheme has good repeatability and engineering application stability.

**Figure 8:**
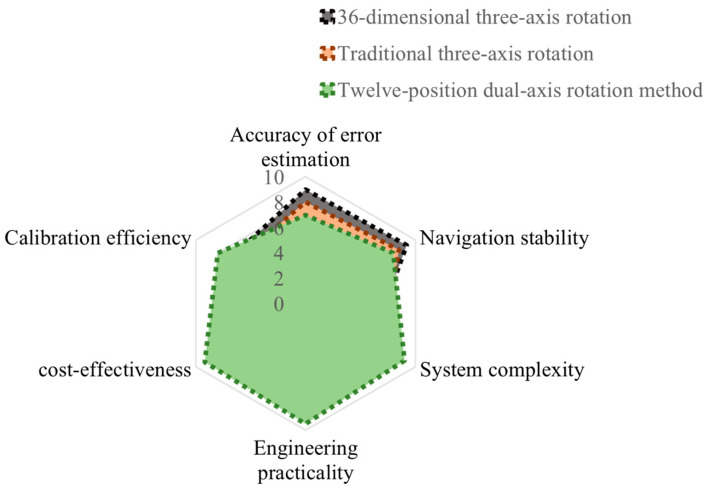
Comparison of radar charts of the three methods.

The 36-dimensional three-axis calibration method in reference [9] and the high-precision three-axis calibration experiment in reference [10] were simulated using the MATLAB 2024a software. The system-level calibration was conducted on the collected real MEMS inertial navigation output data and compared with the rotation modulation method proposed in this paper. A comparison of the error parameter estimation accuracy is shown in Table 4. The influence on the long-term navigation performance of MEMS inertial navigation is shown in Table 5. As shown in the radar chart comparison in Figure 8, for the vast majority of tactical-level MEMS inertial navigation systems, the twelve-position dual-axis calibration method proposed in this paper achieves the most outstanding comprehensive balance in terms of accuracy, stability, cost, practicality, and time and is the most valuable and promising solution for engineering applications and promotion.

## 5. Conclusions

This paper focuses on the calibration and compensation of the initial state system error of micro-electromechanical system (MEMS) inertial navigation. In response to the conflict between accuracy, cost, and dynamic response in traditional methods, a new approach combining engineering practicality and technological innovation is proposed. Considering the nonlinear characteristics of MEMS device errors, the coordinate transformation process of system solution is analyzed by using the small-angle perturbation linearization method. A deterministic error model is constructed to provide precise modeling support for error compensation. A high-precision orthogonal workpiece compensation structure combined with a twelve-position dual-axis calibration method is developed, which can simultaneously calibrate zero bias errors, scale factor errors, and cross-axis coupling errors. Through 50 repeated experiments, the installation error calibration’s accuracy was verified to reach 0.030° ± 0.001°. Compared with the traditional dual-axis system [17], the accuracy of this method was significantly improved by approximately 25% (*p* < 0.001), breaking through the limitations of dual-axis turntables in calibrating cross-coupling errors. This method can meet the core requirements of three-axis system calibration without relying on expensive three-axis turntables and significantly reduces the cost. By precisely calibrating the initial error, the secondary error diffusion problem that occurs over time can be effectively alleviated. The statistical results show that the initial state error of inertial navigation can be stably controlled within 1 μrad. During a one-hour navigation test, the average navigation error of the system was reduced by 90.2% (95% CI: 89.1–91.3%), demonstrating the robustness of the compensation effect of this method.

## Figures and Tables

**Table 1:** Error type parameters of inertial devices.

Error Types	Symbol
Gyro zero bias	$b_{g}$
Gyro scale factor error	$\delta K_{g}$
Gyroscopic cross-coupling error	$M_{g}$
Accelerometer zero bias	$b_{a}$
Accelerometer scale factor error	$\delta K_{a}$

**Table 2:** Zero offset and proportional factor error at Position 1.

Title	*X*-Axis	*Y*-Axis	*Z*-Axis
Zero drift	0.9402	0.0579	0.0010
Scaling factor	1.8347	1.0029	2.0040

**Table 3:** The standard deviation after compensation of the measured values.

Title	*X*-Axis	*Y*-Axis	*Z*-Axis
Standard deviation	0.1170	0.1859	0.7023

**Table 4:** Comparison of estimation accuracies of error parameters.

Error Parameter	36-Dimensional Three-Axis Rotation	Traditional Three-Axis Rotation	Twelve-Position Dual-Axis Rotation Method
**Gyro zero bias** (°/h)	0.498 ± 0.01	0.52 ± 0.05	0.55 ± 0.08
**Gyroscope scale factor** (ppm)	201 ± 5	195 ± 15	195 ± 25
**Installation error** (arcsec)	99 ± 3	95 ± 20	90 ± 30
**Accelerometer zero bias** (mg)	0.501 ± 0.02	0.48 ± 0.1	0.52 ± 0.15

**Table 5:** Long-term navigation performance analysis.

Time	36-Dimensional Three-Axis Rotation	Traditional Three-Axis Rotation	Twelve-Position Dual-Axis Rotation Method
**1 h**	0.062	0.23	0.109
**4 h**	0.25	0.85	0.52
**8 h**	0.51	1.68	1.15
**12 h**	0.78	2.52	1.82

## Data Availability

The raw data supporting the conclusions of this article will be made available by the authors on request.
